# A Comparative Study of Dog- and Cat-Induced Injury on Incidence and Risk Factors among Children

**DOI:** 10.3390/ijerph13111079

**Published:** 2016-11-03

**Authors:** Ying Chen, Yang Gao, Li Zhou, Yafei Tan, Liping Li

**Affiliations:** 1 Center for Injury Prevention Research, Shantou University Medical College, Shantou 515041, China; 13chenying@stu.edu.cn (Y.C.); tanyf1012@163.com (Y.T.); 2 Department of Physical Education, Faculty of Social Sciences, Hong Kong Baptist University, Hong Kong, China; gaoyang@hkbu.edu.hk; 3 Shenzhen Center for Disease Control and Prevention, Shenzhen 518055, China; allicdc@163.com

**Keywords:** dog- and cat-related injury, children, risk factors, China

## Abstract

**Background:** Millions of people are bitten by animals each year, with approximately 90% of the injuries being caused by dogs and cats. However, few studies focus on risk factors of dog- and cat-induced injury in China. Therefore, the objective of this study was to investigate the rate of dog- and cat-induced injury and its potential risk factors. **Methods:** The data were from a population-based cross-sectional study conducted in 2015, with a sample of 9380 children 6–19 years of age from two cities, Shenzhen (large city) and Shantou (mid-sized city), in southern China. Multivariate logistic regression models were used to identify the risk factors of injury by dogs and cats. **Results:** The total rates of dog and cat-induced injury were 15.1% and 8.7% during the lifetime, and 3.4% and 1.7% during the past year, respectively. Dog bites mostly occurred in the dog’s residence (49.4%). Cat scratches were more likely to be inflicted by one’s own cat (47.5%). Children living in suburban and island county had 2.83 times and 2.53 times more dog-related injuries than central urban children, respectively. After stratification by cities, injuries in Shantou were correlated with non-single child families (OR (odds ratios), 1.46; 95% CI (95% confidence interval), 1.09–1.96) and raising cats (OR, 5.34; 95% CI, 3.88–7.35). Those who disliked animals (OR, 0.62; 95% CI, 0.45–0.88) or had good academic performance (OR, 0.46; 95% CI, 0.35–0.60) had lower risk for injury. Injuries in Shenzhen were related to the mother’s educational level (OR, 1.51; 95% CI, 1.11–2.07) and mother being a migrant worker (OR, 2.10; 95% CI, 1.12–3.94). **Conclusions:** Family factors were important to predict dog- and cat-induced injury among children from Shenzhen, and personal factors were closely associated with injury among children form Shantou.

## 1. Introduction

According to the World Health Organization (WHO) reports in 2013, animal bites are a significant cause of morbidity and mortality worldwide [1]. Bites from mammals are common and often require in-patient care [2]. More than two-thirds of injuries are from dogs [3], and cat-induced injuries account for 5%–10% of all animal wounds [4]. Injuries by other animals such as rabbits, monkeys, hamsters, guinea pigs, and mice are rarer [5,6,7]. It is estimated that each year up to 4.7 million Americans suffered from dog bites and approximately 800,000 required medical care [8]. Serious wounds were commonly caused by dog bites, which even created a pathway for rabies transmission [9]. Cat bites/scratch wounds were mostly minor, but cat bites might frequently lead to infections, which occurred in 30%–50% of cases [10]. Complicated consequences of dog- and cat-induced injuries adversely influenced the quality of life of victims and their families, and increased healthcare costs [3,11] and the burden of disease [12] to both families and societies. 

Few previous studies on dog bites have been conducted in Asian countries [13]. Epidemiologists in Taiwan found that children were the most vulnerable population for dog bites [14]. Hong Kong researchers reported similar results, with an average age of dog-bite victims of 11.82 years old [15]. Moreover, children who experienced animal bites were more likely to be seriously injured than adults [16]. The possible reason is that children unknowingly and easily provoke animals because they lack risk awareness [17,18]. They are less likely than adults to recognize the emotions or behavioral signals of animals [19]. In addition, treatments for dog- or cat-induced wounds among children were often inappropriate and delayed [20], which further increased risks for post-traumatic stress syndrome and acute infections. 

Though cats and dogs are the most likely to cause injuries, as family pets they are still becoming more and more popular around the world. Statistical data (2012) from the American Humane Society showed that more than 70% of American households kept a dog or cat [21]. About 78 million dogs and 86 million cats were owned in the USA [16]. Similar situations were also observed in European countries [22]. Previous reports indicated a relatively low ownership rate of dogs and cats in China (30%–40%) [23,24]. However, a rapid rise in ownership has been observed in recent decades, along with the high-speed economic growth [23,24].

Epidemiological characteristics of dog- and cat-induced injury in cities of China are unclear. Though there were many diverse media reports on dog- and cat-induced injury occurrences in both central urban and suburban settings, no research was conducted to investigate the epidemic of such injuries. This study aimed to investigate the rate and patterns of dog- and cat-induced injury and identify the risk factors among children in two different-sized cities in China. Secondly, we made comparisons between the two cities, central urban and suburban areas, and dog- and cat-induced injuries to have a better understanding about and the epidemiological characteristics of the injuries. 

## 2. Methods

### 2.1. Study Population

**Figure 1:**
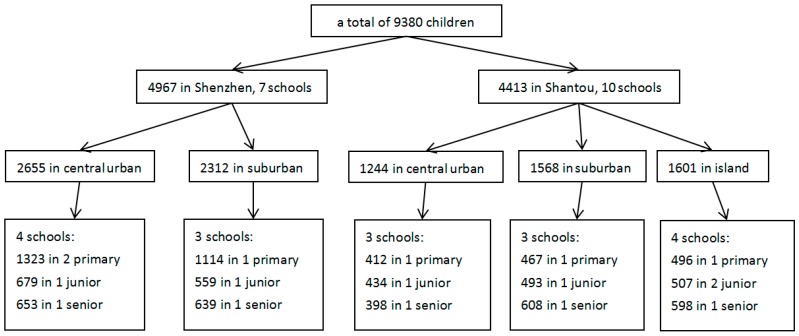
The multistage sampling process.

The present cross-sectional study was conducted among primary and secondary school students (aged 6–19 years) in Shenzhen and Shantou, Guangdong Province, China in April, 2015. We chose Shenzhen and Shantou based on the following considerations. (1) Both are located in southern China but represent different stages of economic development. The two cities vary widely in their gross domestic products (GDPs) and populations, which may play a role in dog- and cat-induced injury. Shenzhen (a well-developed city), neighboring Hong Kong, is one of the top four megacities in China. National migrants in Shenzhen account for 70.59% of the whole population, whilst Shantou (a developing city) is a relatively conservative and historical city, with its population mostly consisting of original residents. (2) We had conducted several studies on child injury and built up sound networks with local schools in both of the cities [25,26]. A multistage sampling method was applied to ensure a representative sample within each city (Figure 1). Firstly, a total of 17 schools, including 6 primary schools and 6 junior and 5 senior secondary schools, were randomly selected as objects of study. We numbered all the schools of the two cities in different districts (divided into central urban and suburban) and selected several primary schools, junior schools, and senior schools through random sampling to distribute as evenly as possible in each district and grade. Secondly, all students in grades 1–11 (age 6–19) of the study schools were invited to participate in the study. Written informed consent forms were obtained from their parents in advance. This study was approved by the Ethics Committee of the Shantou University Medical College (No. SUMC-2015-41).

### 2.2. Data Collection and Measures

A self-administered questionnaire was used to collect data. We had performed a test-retest before the investigation: we gave a pretest to 45 students from a class, and we retested with the same questionnaire 15 days later among the same individuals. The results showed that the questionnaire had good reliability and the correlation coefficient ranged from 0.786 to 0.851 (*p* < 0.01). Exploratory factor analysis was performed to assess the construct validity of the questionnaire, and the results showed that it had good construct validity. The data we collected involved sociodemographics, including personal factors (cities, gender, age, living area, single-child family, personality, interests in animals, interests in physical exercise, academic performance, and keeping dogs and cats) and family factors (parents’ educational level, parents’ occupation, migrant workers for parents, marital status of parents, average monthly income), and characteristics of dog- and cat-induced injury experiences in their lifetime (defined as lifetime injury) and in the past 12 months (defined as past-year injury). A dog- and cat-induced injury episode was defined as any nonfatal physical damage to the child’s body caused by a dog or cat, and children could recall it precisely (e.g., bites, scratches, falls caused by dog or cat). 

Children in grades 4–11 (aged 10–19) completed their questionnaires in the classroom, with assistance from trained investigators if needed. Younger students (those aged 6–9) took the questionnaires home and completed them with their parents. All completed questionnaires were checked immediately after collection for accuracy and completeness. Follow-ups were performed if necessary. 

### 2.3. Statistical Analysis

Chi-square test was used to test between-group differences. Univariate and multivariate logistic regression analyses were performed to identify the risk factors for injury. All the demographics, and personal and family factors, were analyzed using forward stepwise regression with criteria of *p* < 0.10 for entry and *p* < 0.20 for removal. Lifetime injury was defined as the dependent variable. Odds ratios (OR) and 95% confidence interval (95% CI) were computed to assess the associations between the risk factors and injuries. A significance level of 0.05 was adopted. All the analyses were carried out using SPSS version 19.0 software (SPSS Inc., Chicago, IL, USA).

## 3. Results

### 3.1. Demographic Characteristics of Participants

A total of 9380 children aged 6–19 years, consisting of 4739 boys (50.5%) and 4641 girls (49.5%), participated in the study, with a participation rate of 96.9%. The characteristics of the participants are shown in Table 1. We also listed the statistics of populations, areas, and GDPs of the two cities (in 2014) in Table 1. About 15.5% of the families currently raised a dog (8.1%) or cat (7.4%), and 32.2% of families raised a dog or cat once. In Shenzhen, 9.9% of families had a dog, which was higher (*p* < 0.001) than Shantou (6.0%). In contrast, 11.4% families raised a cat in Shantou, but only 3.9% in Shenzhen (*p* < 0.001).

### 3.2. The Rate of Dog- and Cat-Related Injuries among Children in Two Cities

As shown in Table 2, there were 1413 (15.1%) individuals who had sustained injuries from dogs, such as bites, scratches, falls, and both bites and scratches. The predominant injuries were bites (57.5%) on hands (34.9%) and legs (27.8%). Compared with other types of injuries, bites were common injuries caused by dogs (57.5%), whereas scratches (77.1%) were usually caused by cats mainly on the hand (62.7%) (*p* < 0.001). Children of Shenzhen had higher dog-related injuries rate during their lifetime (18.1% vs. 11.7%, *p* < 0.001) and also had higher rate in past year (4.2% vs. 2.6%, *p* < 0.001) than those in Shantou; while for cat-related injuries, children of Shantou had higher rate of injury both in lifetime and past year (9.6% vs. 7.9%, *p* = 0.003) than those in Shenzhen, but there was no significance in the past-year injury (2.0% vs. 1.5%, *p* = 0.08). 

Characteristics of injuries are shown in Table 3. Dog-related injuries were mainly caused by other families’ dogs (49.4%), but more cat-related injuries were due to the cat owned by the victims (47.5%) (*p* < 0.001).

### 3.3. Characteristics and Risk Factors of Children Injured by Dog and Cat

Table 4 showed the risk factors associated with injuries by dogs and cats, as determined by univariate logistic regression analysis. There was a significant difference in the distribution among cities, gender, age, living area, single-child family, personality, interest in animals, academic performance, parents’ educational level, migrant workers for parents, average monthly income, and keeping pets between children with and without a dog- or cat-related injury. However, significant relationships were maintained only for the mother’s educational level, migrant workers for parents, and keeping different pets when using the multivariate logistic regression analysis. 

### 3.4. Risk Factors Associated with Injured Children from Different-Sized Cities

In order to explore the risk factors associated with injured children for differently sized cities, a hierarchical multiple regression analysis was conducted (Table 5). For children in Shenzhen, living in a suburban area, being in the age range 10–15, and having average and poor school performance were significantly associated with dog- and cat-induced injury. After stratification by different animals, children from suburban areas and in the age range 10–15 were vulnerable to dog- and cat-induced injury, and those whose mothers were out working were associated with dog-induced injury. Having a mother with a lower education degree was a protective factor for children from cat-induced injury. For children in Shantou, having a brother or sister, being fond of pets, average and poor school performance, and once having or currently keeping pets were associated with dog- and cat-induced injury. Moreover, children who lived on an island were easily injured by dogs. Compared to children in the age range 6–9, children of higher ages were more vulnerable to injury by cats. 

## 4. Discussion

In our study, we investigated 9380 children and presented results of the incidence, characteristics, and associated risk factors of dog- and cat-induced injury among children in two differently sized cities: Shenzhen (large city) and Shantou (mid-sized city) in southern China. To our knowledge, this is the first epidemiological survey, with a large and population-based sample, that explores the relationship between city size and dog/cat-caused injuries, which includes all kinds of possible injuries by dogs and cats, and compares the characteristics and associated risk factors of dog- and cat-induced injury in one survey.

There were significant differences between the two cities in the notion of raising pets. According to our interviews with residents in pilot investigations, we found that people preferred keeping cats to dogs in Shantou: the reason was probably the rat infestation in this city in both central urban and suburban areas. On the other hand, in Shenzhen, most central urban residents chose a dog as a pet, particularly in suburban areas to guard houses. As results showed, there existed a large difference between the two cities in the percentage of cats and dogs raised and the types of attacking animal. 

Through our study, we found that the total rate of dog-induced injury during one’s lifetime (15.1%) was lower than the reported rate in the U.S. (35%–46% [28,29]), but higher than Jiangxi province (1.93% [30]) in China. Considering the different definitions, regions, and ages of victims in other studies, we cannot make accurate comparisons. However, it seems clear that dog-related injuries are higher than cat-related injuries, regardless of region and victim age [31,32,33]. We also support previous studies showing that boys had a higher risk of animal-induced injury than girls [20,34,35]. This is probably because boys have higher levels of sensation seeking [36] and are more impulsive than girls [37]. The children with poor academic performance had higher risk for injury compared with those who had good academic performance, possibly because those spending less time on their studies were playful and curious of other things, like making fun of animals.

We found that injury by dogs, in the majority of cases, occurred where the dog lives, similar to a previous dog bite survey [38,39], whereas most children attacked by cats were in their own home and attacked by their pet cats. Dogs serve as house guards and attack strangers who visit the house, but cats are closer to their owners, and are embraced or even kissed. Such behavior toward cats is more likely to lead to intentional or unintentional bites or scratches. Similarly, previous studies in developed countries also suggested that wounds were common in arms (hands) by cats, and legs (feet) by dogs [40,41,42], although other studies indicated that dog bites could occur mostly on a child’s face, because a standing dog can be the same height as children [39]. However, in our study, all victims were school-aged children, and were generally taller than a dog or cat.

Moreover, the populations in the two cities are tremendously different, since non-native residents, who are mostly migrant workers, occupy a large proportion (70.59%) of the population in Shenzhen, and mostly live in the suburbs where the degree of development is far worse than central urban. Meanwhile, there is little central-suburban variance in Shantou, which is a relatively conservative and historical city, and the population is mostly comprises original residents. Children living in the large city Shenzhen or an island in Shantou have much higher risks for dog-related injuries than in the mid-size city of Shantou. Multivariate logistic regression analyses, stratified by attacking animals, indicated that being accompanied by parents was crucial for prevention of injuries by a dog. Keeping pets, as a factor for dog- and cat-related injuries, was significant only in Shantou, and probably indicates that family pet-raising habits have a great influence on children. Furthermore, our findings suggested that personal factors, such as having a brother or sister, poor academic performance, being fond of animals, and raising pets were associated with a higher risk of animal-related injury in Shantou. In Shenzhen, however, family factors such as living in a suburban area, lower education of the mother, and parents working outside the home were the main risk factors for children. Migrant families in large cities often have no fixed residence for the long term, moving frequently for work. Moreover, having one or both parents working in a central urban area, and leaving the children alone or with grandparents or guardians, could reduce the quality of care for children. This may be a possible explanation for children with migrant parents having a high risk for dog-induced injury, and could have important implications for parental accompaniment of suburban children. In addition, parental factors (migrant workers for parents, mother’s educational level) were important to predict dog- and cat-induced injury among children from Shenzhen, and personal factors (academic performance, single-child, raising pets) were closely associated with injury among children from Shantou. Generally, animal injuries in children were associated with contact behavior or affection for animals in the results for Shantou. However, for Shenzhen, a city with large populations of immigrants, family factors like family care and companionship for children seemed especially important. 

Our study has some limitations. First, there was recall bias in the cross-sectional study, since the outcome measurement was based on self-reporting. Consequently, the information was probably susceptible to self-perception and interpretation. Second, the participants who were under 9 years old answered the questionnaire with their parents’ guidance, which might underestimate the problem, because the injured children might fear being blamed by their parents. In future research, a return survey will be conducted to check the information of respondents to measure and control recall bias, using better designs such as prospective or case-crossover studies. Despite these limitations, we systematically analyzed the rate and related factors for dog- and cat-induced injuries among children in large and mid-sized cities of China.

## 5. Conclusions

In summary, dog- and cat-induced injuries have turned into a public health problem among children, with a high incident rate in Shenzhen and Shantou City, China. We compared characteristics of dog- and cat-related injury and risk factors in the two different cities. We found that children in the age range 10–14, living in suburban areas, and having poor academic performance were risk groups. Children with mothers who go out for work and have a low educational level are at high risk for dog-induced injury in Shenzhen. We suggested that company and protection from parents is important for left-behind children to prevent animal injury in large cities. In Shantou, a mid-sized city, being fond of animals and having dogs and cats as pets were risk factors for children. Here, the strategies may include educating students about potential risks of animal injury and how to safely contact and communication with dogs and cats.

## Figures and Tables

**Table 1:** Characteristics of children in a sample of 9380 in Shenzhen and Shantou, China, and city statistics information.

Characteristics		Shenzhen (*N* = 4967)	Shantou (*N* = 4413)	Total (*N* = 9380)
		*n* (%)	*n* (%)	*n* (%)
Age Group (Years)	6~	924 (18.6)	426 (9.7)	1876 (20.0)
	10~	1179 (23.7)	601 (13.6)	3132 (33.4)
	14~	1389 (28.0)	1495 (33.9)	2827 (30.1)
	16–19	1475 (29.7)	1891 (42.8)	1545 (16.5)
Gender	Boy	2681 (54.0)	2058 (46.6)	4739 (50.5)
	Girl	2286 (46.0)	2355 (53.4)	4641 (49.5)
Living Area	Central Urban	2655 (53.5)	1244 (28.2)	3899 (41.6)
	Suburban	2312 (46.5)	1568 (35.5)	3880 (41.4)
	Island County	-	1601 (36.3)	1601 (17.1)
Raising Dogs and Cats	Never	2824 (56.9)	2083 (47.2)	4908 (52.3)
	Ever but Not Now	1454 (29.3)	1561 (35.4)	3015 (32.2)
	Currently Cat	194 (3.9)	504 (11.4)	698 (7.4)
	Currently Dog	495 (9.9)	265 (6.0)	760 (8.1)
City Statistics *	Population (Million)	10.78	5.53	-
	Locals	3.17 (29.41)	5.47 (98.92)	-
	Migrants	7.61 (70.59)	0.058 (1.08)	-
	Area (km^2^)	1991.64	2064.73	-
	GDP (Billion RMB)	1600.198	171.651	-

* 2014 statistical yearbook [27], GDP = gross domestic products, RMB = renminbi.

**Table 2:** Incidence and the relationship between the cities and dogs and cats in a sample of 9380 students in Shenzhen and Shantou, China (2015).

*N* = 9380	Dog		*p*-Value	Cat		*p*-Value
	*n*	%		*n*	%	
Lifetime Injury	-	-	<0.001	-	-	0.003
Shenzhen	898	18.1	-	392	7.9	-
Shantou	515	11.7	-	425	9.6	-
Total	1413	15.1	-	817	8.7	-
Past-Year Injury	-	-	<0.001	-	-	0.080
Shenzhen	208	4.2	-	74	1.5	-
Shantou	114	2.6	-	87	2.0	-
Total	322	3.4	-	161	1.7	-

**Table 3:** Characteristics of dog- and cat-related injuries among children in Shenzhen and Shantou, China (2015).

Characteristics	Dog (*N* = 1413)		*p*-Value	Cat (*N* = 817)		*p*-Value
	*n*	%		*n*	%	
Type of Injury	-	-	<0.001	-	-	<0.001
Bites	812	57.5	-	102	12.5	-
Scratches	360	25.5	-	630	77.1	-
Falls	125	8.8	-	33	4.0	-
Both Bites and Scratches	116	8.2	-	52	6.4	-
Ownership of Involved Animal	-	-	<0.001	-	-	<0.001
Own Family’s	425	30.3	-	386	47.5	-
Other Family’s	693	49.4	-	241	29.6	-
Stray Dog/Cat	131	9.3	-	114	14.0	-
Unknown	154	11.0	-	72	8.9	-
Injured Body Part	-	-	<0.001	-	-	<0.001
Hand	469	34.9	-	501	62.7	-
Arm	148	11.0	-	80	10.0	-
Foot	256	19.1	-	96	12.0	-
Leg	374	27.8	-	59	7.4	-
Head/Face/Neck	45	3.4	-	51	6.4	-
Trunk	52	3.9	-	12	1.5	-

**Table 4:** Unadjusted and adjusted associations between the demographic variables and injuries stratified by dog and cat in a sample of 9380 children in Shenzhen and Shantou, China (2015).

Variables	Unadjusted OR (95% CI)						Adjusted OR (95% CI)					
	Dog			Cat			Dog			Cat		
	*p*-Value	OR	95% CI	*p*-Value	OR	95% CI	*p*-Value	OR	95% CI	*p*-Value	OR	95% CI
City	-	-	-	-	-	-	-	-	-	-	-	-
Shenzhen	<0.001	1.67	1.49–1.88	0.003	1.24	1.08–1.44	<0.001	1.88	1.57–2.24	-	-	-
Shantou	-	1.00	-	-	1.00	-	-	1.00	-	-	-	-
Gender	-	-	-	-	-	-	-	-	-	-	-	-
Girls	-	1.00	-	-	1.00	-	-	-	-	-	-	-
Boys	0.024	0.88	0.78–0.98	0.207	1.10	0.95–1.27	-	-	-	-	-	-
Age (Years)	-	-	-	-	-	-	-	-	-	-	-	-
6~	-	1.00	-	-	1.00	-	-	1.00	-	-	1.00	-
10~	<0.001	2.55	2.04–3.17	<0.001	3.43	2.46–4.77	<0.001	2.58	1.99–3.33	<0.001	2.79	1.95–4.00
14~	<0.001	2.00	1.62–2.47	<0.001	3.23	2.35–4.44	<0.001	2.03	1.57–2.61	<0.001	2.57	1.81–3.64
16–19	<0.001	1.66	1.30–2.05	<0.001	2.61	1.90–3.59	<0.001	1.60	1.23–2.07	0.001	1.85	1.30–2.65
Living Area	-	-	-	-	-	-	-	-	-	-	-	-
Central Urban	-	1.00	-	-	1.00	-	-	1.00	-	-	1.00	-
Suburban	<0.001	3.16	2.76–3.62	<0.001	1.47	1.25–1.72	<0.001	2.83	2.38–3.35	<0.001	1.48	1.23–1.79
Island County	<0.001	1.66	1.38–2.00	0.003	1.37	1.11–1.68	<0.001	2.53	1.98–3.24	0.013	1.35	1.06–1.70
Single-Child Family	-	-	-	-	-	-	-	-	-	-	-	-
Yes	-	1.00	-	-	1.00	-	-	-	-	-	-	-
No	<0.001	1.66	1.43–1.93	0.064	1.18	0.99–1.41	-	-	-	-	-	-
Personality	-	-	-	-	-	-	-	-	-	-	-	-
Introverted	-	1.00	-	-	1.00	-	-	-	-	-	-	-
Normal	0.004	1.31	1.09–1.57	0.162	1.18	0.94–1.48	-	-	-	-	-	-
Extroverted	0.188	1.09	0.96–1.25	0.653	0.96	0.82–1.14	-	-	-	-	-	-
Interest in Animal	-	-	-	-	-	-	-	-	-	-	-	-
Dislike	-	1.00	-	-	1.00	-	-	1.00			1.00	-
Normal	0.001	1.47	1.16–1.87	0.560	1.09	0.81–1.47	0.002	0.65	0.49–0.85	0.677	1.08	0.76–1.52
Like	<0.001	1.59	1.26–2.00	0.001	1.62	1.21–2.16	0.005	0.43	0.28–0.57	0.006	1.60	1.14–2.25
Academic Performance	-	-	-	-	-	-	-	-	-	-	-	-
Good	<0.001	0.43	0.38–0.50	<0.001	0.63	0.52–0.76	-	-	-	0.002	0.72	0.58–0.89
Average	<0.001	0.70	0.60–0.80	0.348	0.91	0.76–1.10	-	-	-	0.887	0.99	0.79–1.22
Poor	-	1.00	-	-	1.00	-	-	-	-	-	1.00	-
Father’s Educational Level	-	-	-	-	-	-	-	-	-	-	-	-
Primary School or Less	<0.001	2.02	1.62–2.51	<0.001	1.85	1.41–2.42	-	-	-	-	-	-
Junior High School	<0.001	1.83	1.53–2.19	0.001	1.48	1.18–1.85	-	-	-	-	-	-
Senior High School	0.006	1.31	1.08–1.58	0.015	1.34	1.06–1.69	-	-	-	-	-	-
University or Above	-	1.00	-	-	1.00	-	-	-	-	-	-	-
Mother’s Educational Level	-	-	-	-	-	-	-	-	-	-	-	-
Primary School Or Less	<0.001	2.07	1.67–2.57	0.014	1.38	1.07–1.78	-	-	-	-	-	-
Junior High School	<0.001	1.96	1.60–2.41	0.007	1.39	1.09–1.77	-	-	-	-	-	-
Senior High School	0.006	1.36	1.09–1.69	0.130	1.22	0.94–1.58	-	-	-	-	-	-
University or Above	-	1.00	-	-	1.00	-	-	-	-	-	-	-
Migrant Parents for Work	-	-	-	-	-	-	-	-	-	-	-	-
No Parents Go Out	-	1.00	-	-	1.00	-	-	-	-	-	-	-
Father Go Out	0.655	1.05	0.86–1.28	0.662	0.95	0.74–1.22	-	-	-	-	-	-
Mother Go Out	<0.001	2.25	1.51–3.37	0.802	1.08	0.59–1.97	-	-	-	-	-	-
Both Parents Go Out	<0.001	2.13	1.83–2.47	0.987	1.00	0.80–1.24	-	-	-	-	-	-
Average Monthly Income	-	-	-	-	-	-	-	-	-	-	-	-
Low	0.002	1.35	1.12–1.63	0.112	1.21	0.96–1.54	-	-	-	-	-	-
Average	0.205	1.14	0.93–1.41	0.596	1.07	0.83–1.39	-	-	-	-	-	-
High	-	1.00	-	-	1.00	-	-	-	-	-	-	-
Raising Dogs and Cats	-	-	-	-	-	-	-	-	-	-	-	-
Never	-	1.00	-	-	1.00	-	-	-	-	-	1.00	-
Ever but Not Now	0.014	1.18	1.03–1.34	<0.001	1.50	1.27–1.77	-	-	-	0.003	1.33	1.10–1.61
Currently Cat	0.962	0.99	0.79–1.26	<0.001	3.82	3.08–4.73	-	-	-	<0.001	3.32	2.59–4.25
Currently Dog	<0.001	2.53	2.12–3.03	0.009	1.44	1.10–1.89	-	-	-	0.505	1.11	0.82–1.51

CI, confidence interval; OR, odds ratio.

**Table 5:** Characteristics of dog- and cat-related injury among children in Shenzhen and Shantou, China, multivariate logistic regression analyses stratified by cities of residence (2015).

Variables	Shenzhen OR (95% CI)						Shantou OR (95% CI)					
	Dog			Cat			Dog			Cat		
	*p*-Value	OR	95% CI	*p*-Value	OR	95% CI	*p*-Value	OR	95% CI	*p*-Value	OR	95% CI
Age (years)	-	-	-	-	-	-	-	-	-	-	-	-
6~	-	1.00	-	-	1.00	-	-	-	-	-	1.00	-
10~	<0.001	3.06	2.27–4.12	<0.001	3.60	2.30–5.63	-			0.027	1.99	1.08–3.66
14~	<0.001	2.57	1.90–3.48	<0.001	3.55	2.26–5.56	-	-	-	0.012	2.08	1.17–3.68
16~19	0.018	1.46	1.07–2.00	0.030	1.69	1.05–2.71	-	-	-	0.044	1.81	1.02–3.23
Living Area	-	-	-	-	-	-	-	-	-	-	-	-
Central Urban	-	1.00	-	-	1.00	-	-	1.00	-	-	-	-
Suburban	<0.001	4.57	3.68–5.66	<0.001	2.49	1.90–3.25	0.040	1.36	1.02–1.83	-	-	-
Island county	-	-	-	-	-	-	0.008	1.45	1.10–1.92	-	-	-
Single-Child Family	-	-	-	-	-	-	-	-	-	-	-	-
Yes	-	-	-	-	-	-	-	1.00	-	-	-	-
No	-	-	-	-	-	-	0.012	1.46	1.09–1.96	-	-	-
Interest in Animal	-	-	-	-	-	-	-	-	-	-	-	-
Dislike	-	-	-	-	-	-	0.015	0.59	0.38–0.90	0.006	0.62	0.45–0.88
Normal	-	-	-	-	-	-	0.420	0.91	0.73–1.14	<0.001	0.67	0.56–0.80
Like	-	-	-	-	-	-	-	1.00	-	-	1.00	-
Academic Performance	-	-	-	-	-	-	-	-	-	-	-	-
Good	0.006	0.73	0.58–0.92	0.185	0.81	0.59–1.11	<0.001	0.46	0.35–0.60	0.027	0.70	0.52–0.96
Average	0.110	0.83	0.66–1.04	0.432	1.13	0.83–1.54	0.378	0.89	0.68–1.16	0.452	0.89	0.65–1.21
Poor	-	1.00	-	-	1.00	-	-	1.00	-		1.00	-
Mother’s Educational Level	-	-	-	-	-	-	-	-	-	-	-	-
Primary School or Less	0.010	1.51	1.11–2.07	-	-	-	-	-	-	-	-	-
Junior High School	0.115	1.26	0.95–1.68	-	-	-	-	-	-	-	-	-
Senior High School	0.624	0.93	0.68–1.26	-	-	-	-	-	-	-	-	-
University or Above	-	1.00	-	-	-	-	-	-	-	-	-	-
Migrant Parents for Work	-	-	-	-	-	-	-	-	-	-	-	-
No Parents Go Out	-	1.00	-	-	-	-	-	1.00	-	-	-	-
Father Go Out	0.200	1.24	0.89–1.73	-	-	-	0.373	0.86	0.61–1.21	-	-	-
Mother Go Out	0.021	2.10	1.12–3.94	-	-	-	0.307	1.56	0.67–3.63	-	-	-
Both Parents Go Out	0.065	1.22	0.99–1.51	-	-	-	0.028	1.60	1.05–2.43	-	-	-
Raising Dogs and Cats	-	-	-	-	-	-	-	-	-	-	-	-
Never	-	-	-	-	-	-		1.00	-	-	1.00	-
Ever but Not Now	-	-	-	-	-	-	0.195	1.17	0.92–1.49	<0.001	1.85	1.39–2.46
Currently Cat	-	-	-	-	-	-	0.254	0.79	0.54–1.18	<0.001	5.34	3.88–7.35
Currently Dog	-	-	-	-	-	-	<0.001	2.76	1.93–3.94	0.277	1.34	0.79–2.27
